# A quantitative real-time RT-PCR assay for mature *C. albicans *biofilms

**DOI:** 10.1186/1471-2180-11-93

**Published:** 2011-05-06

**Authors:** Zhihong Xie, Angela Thompson, Helena Kashleva, Anna Dongari-Bagtzoglou

**Affiliations:** 1 Division of Periodontology, School of Dental Medicine, University of Connecticut, Farmington, Connecticut, USA

## Abstract

**Background:**

Fungal biofilms are more resistant to anti-fungal drugs than organisms in planktonic form. Traditionally, susceptibility of biofilms to anti-fungal agents has been measured using the 2,3-bis(2-methoxy-4-nitro-5-sulfophenyl)-2H-tetrazolium-5-carboxyanilide (XTT) assay, which measures the ability of metabolically active cells to convert tetrazolium dyes into colored formazan derivatives. However, this assay has limitations when applied to high *C. albicans *cell densities because substrate concentration and solubility are limiting factors in the reaction. Because mature biofilms are composed of high cell density populations we sought to develop a quantitative real-time RT-PCR assay (qRT-PCR) that could accurately assess mature biofilm changes in response to a wide variety of anti-fungal agents, including host immune cells.

**Results:**

The XTT and qRT-PCR assays were in good agreement when biofilm changes were measured in planktonic cultures or in early biofilms which contain lower cell densities. However, the real-time qRT-PCR assay could also accurately quantify small-medium size changes in mature biofilms caused by mechanical biomass reduction, antifungal drugs or immune effector cells, that were not accurately quantifiable with the XTT assay.

**Conclusions:**

We conclude that the qRT-PCR assay is more accurate than the XTT assay when measuring small-medium size effects of anti-fungal agents against mature biofilms. This assay is also more appropriate when mature biofilm susceptibility to anti-fungal agents is tested on complex biological surfaces, such as organotypic cultures.

## Background

Microbial biofilms have an innate resistance to antimicrobials and immune attack and have been recently linked to many recalcitrant or recurrent infections [1-3]. The ability of *C. albicans *to form biofilms on prosthetic devices and mucosal surfaces is believed to be intimately associated with its ability to trigger systemic or mucosal infection [4-6]. Therefore the development of novel anti-biofilm agents is of paramount importance in the treatment or prevention of these infections.

Susceptibility of *Candida *biofilms to anti-fungal agents is frequently measured using colorimetric assays that estimate metabolic activity of viable cells residing in biofilms [2,6,7]. Such assays have also been widely used to assess viable cell numbers [8-16]. In these assays metabolically active cells convert tetrazolium dyes into colored formazan derivatives that can be measured by a multi-well scanning spectrophotometer [9,14,16-21]. A key component of one of the formazan assays is sodium salt of 2,3-bis(2-methoxy-4-nitro-5-sulfophenyl)-2H-tetrazolium-5-carboxyanilide, or XTT. Mitochondrial dehydrogenases of viable cells cleave the tetrazolium ring of XTT yielding water-soluble orange formazan. The bioreduction of XTT is inefficient and can be potentiated by addition of an electron-coupling agent such as phenazine methosulfate [9,13,16,17,19,22], menadione [2,13,16,19,22] or coenzyme Q0 (CoQ) [15,20,23].

The XTT assay has been used under various conditions for viability assessment of different organisms including mammalian cells, bacteria and fungi [19,24]. Its wide-spread use is due to the fact that it is simple, fast, and does not require highly specialized equipment other than a spectrophotometer. However, it is accurate only if there is a linear relationship between cell metabolic activity (or cell number) and colorimetric signal. Thus, for the assay to be quantitative, it is important to optimize several key experimental parameters (such as cell number, concentration of XTT, type and concentration of electron-coupling agent) for every organism and every experimental condition [5,12,13,15]. Assay optimization can be more challenging in mature biofilms since metabolic activity and viable cell number may not be linearly related [12,13]. In addition, colony counting methods of viability assessment are not appropriate for mature biofilms since they consist of multicellular hyphal organisms encased in a polysaccharide matrix, which hinders single cell colony growth. Given these facts we sought to critically examine the limitations of the XTT assay in measuring metabolic changes in mature biofilms and develop a molecular assay based on PCR for biofilm viability estimates that would overcome these limitations.

## Results

**Figure 1:**
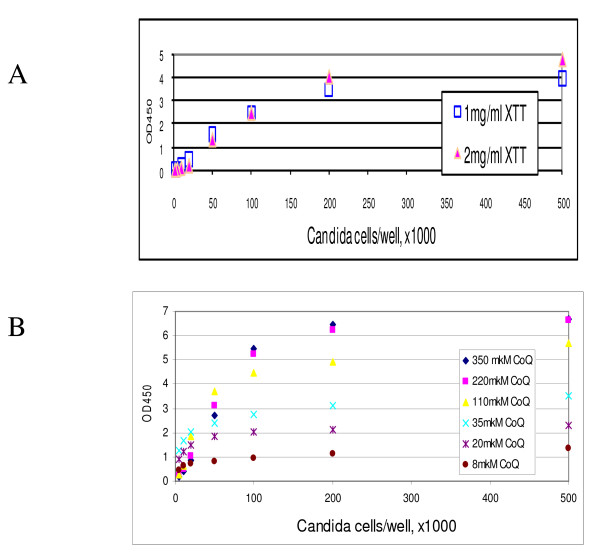
**Effect of XTT assay parameters in the assessment of *C. albicans *metabolic activity**. Overnight planktonic cultures of *C. albicans *yeast cells were seeded at 10^3^-5 × 10^5 ^cells per well (30 mm^2 ^well surface area) and XTT assay was performed as described. (A) Relationship between OD450 and *Candida *cell density at two different XTT concentrations. (B) Effect of CoQ concentration on the linearity range. A representative of three independent experiments is shown.

We first tried to optimize the XTT assay for a wide range of *Candida *cell densities, which would represent different stages of biofilm growth. As shown in Figure 1A-B overall, a linear relationship between the OD450 signal and yeast cell number was observed only when yeast did not exceed 1 × 10^5 ^cells per well. Above this cell density, significant changes in yeast cell number (2-fold or greater) resulted in very small or undetectable differences in OD450 values. This suggests that the XTT assay would be of limited value in mature biofilms, since *C. albicans *biofilms are frequently started by seeding ≥1 × 10^5 ^yeast cells per well, in 96 well plates, and grown for 48h or longer for biofilms to mature [2,6,28].

Increasing the concentration of XTT up to 2 mg/ml (since XTT maximum solubility in water is 2.5 mg/ml) did not result in a change in OD450 when the seeding yeast cell number was equal to or lower than 1 × 10^5 ^cells per well (Figure 1A). With yeast cell numbers higher than 1 × 10^5 ^cells per well, increasing the concentration of XTT resulted in higher OD450 values, which extended the linearity range only up to 2 × 10^5 ^cells per well. This suggests that XTT solubility and final concentration are limiting factors in this reaction, especially when large numbers of yeast cells are used to start biofilms.

We also investigated if varying the concentration of the electron-coupling agent CoQ (8-350 μM) would allow us to extend the linearity range of the XTT signal. XTT conversion rates were slower at lower concentrations of CoQ, generating flat slopes (Figure 1B). However, we found that increasing the concentration of CoQ would not increase the linearity range (Figure 1B). Reading the plates at 490 nm as opposed to 450 nm or increasing the XTT reaction time to 3 hours still did not improve the linearity range (data not shown), since reaction time in higher cell densities (>10^6 ^cells/well) was typically very fast (less than 10 min). Collectively, these data suggest that the XTT assay cannot be adequately optimized to accommodate the cell numbers present in mature biofilms.

To further address this issue we developed a molecular assay for biofilm assessment based on quantitation of *EFB1 *gene transcripts in *Candida *cells, since the EF-1β protein is critical in regulating cell protein translation rates and growth [25]. The *EFB1 *primer pairs specifically amplified PCR products of the predicted size (136 bp) from *C. albicans *cDNA and gave no PCR product when tested with HL-60 cell cDNA (data not shown). To generate standard curves amplification of serially diluted plasmid pEFB was monitored by fluorescence versus cycle number curves. The assay could detect 1 fg of pEFB, which is equivalent to 224.37 copies of pEFB.

**Figure 2:**
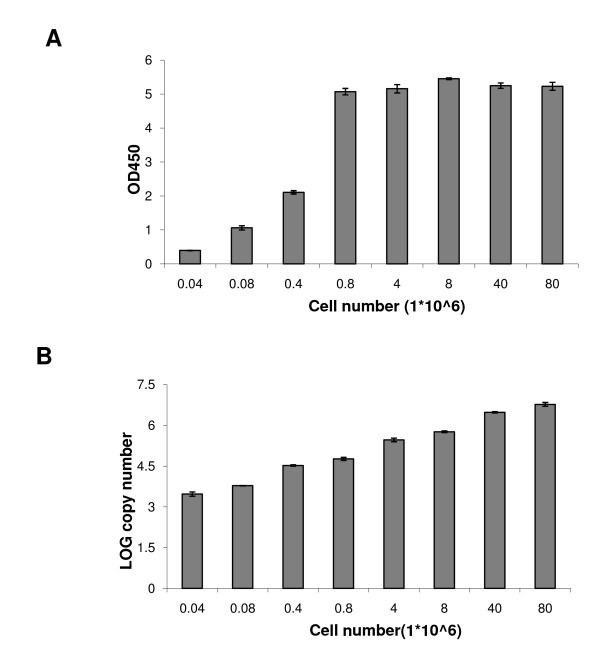
**Comparison of the XTT and real-time RT-PCR assay signals with different seeding cell densities**. Cells were seeded at densities ranging between 4 × 10^4^-8 × 10^7 ^cells per 30 mm^2 ^of well surface area. (A) XTT assay data, expressed as OD450 units, corresponding to each cell density. (B) Quantitative Real-Time RT-PCR assay data, expressed as the mean log10 copy numbers of the *EFB1 *transcript corresponding to each cell density. Means and standard deviations of three independent experiments are shown.

Comparison of the two assays in quantifying viable cells at a wide range of seeding cell densities showed that in contrast to the XTT assay, which gave a flat colorimetric signal for cell densities exceeding 4 × 10^5^/30 mm^2 ^of surface area, the new assay was able to quantify cells at densities up to 8 × 10^7^/30 mm^2 ^(Figure 2A-B). In fact, two fold differences in viable cells were accurately quantified at seeding densities ranging between 4 × 10^4^-8 × 10^7^/30 mm^2 ^with the qRT-PCR assay (Figure 2B).

**Figure 3:**
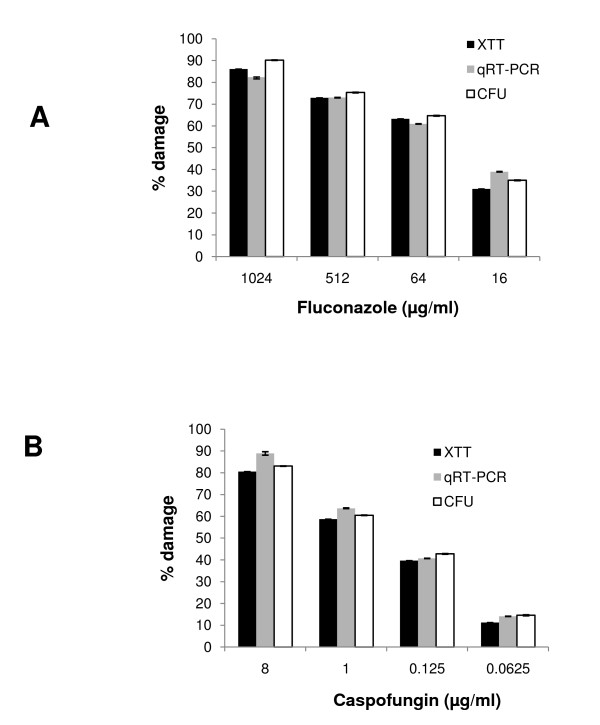
**Comparison of the viable colony counts (CFU), XTT and real-time RT-PCR assays in testing susceptibility of planktonic cells to fluconazole (A) and caspofungin (B)**. Candida yeast cells were exposed to a wide range of concentrations of the antifungal drugs for 24 hours, followed by the CFU, XTT, or *EFB1 *qRT-PCR assays. Error bars represent SD of triplicate experiments.

**Figure 4:**
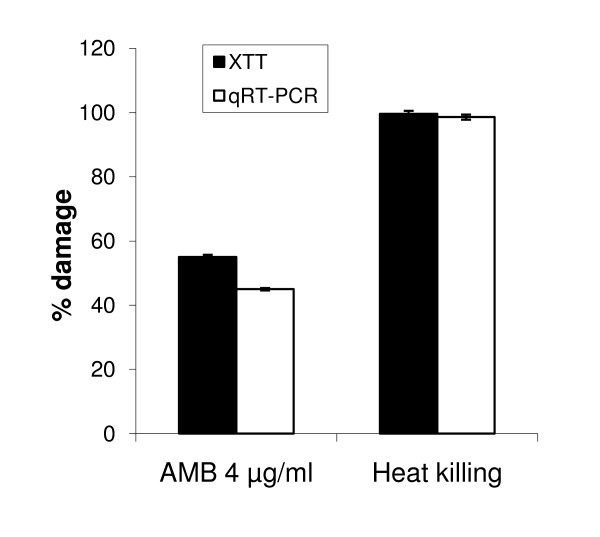
**Comparison of the XTT and qRT-PCR assays in the assessment of early biofilm toxicity**. *Candida *cells were seeded at 10^5 ^cells per 30 mm^2 ^of well surface area and were incubated for 3 h at 37°C prior to exposure to amphotericin B (4 μg/ml, 4 h) or heat (100°C, 1 h). Dark bars represent % biofilm damage, calculated from change in mean OD450 values (XTT assay) and light bars represent % damage calculated from change in mean *EFB1 *transcript copy numbers (qRT-PCR assay) under each experimental condition, as described in the text. Error bars represent standard deviations from three independent experiments.

To further assess the accuracy of the qRT-PCR assay we compared it to viable colony counts, as well as to the XTT assay, in detecting viability changes in planktonic cells triggered by fluconazole or caspofungin. As shown in Figure 3, the qRT-PCR assay could accurately quantify a dose-dependent antifungal drug toxicity in planktonic cells and was in good agreement with the XTT and CFU assays (Figure 3). Our data also show that the XTT and qRT-PCR assays were in good agreement in quantifying toxicity in early biofilms triggered by amphotericin B, whereas organisms killed by heat produced no signal in the XTT or qRT-PCR assay (Figure 4). The latter was confirmed by the absence of CFU's in Sabouraud agar plates.

**Figure 5:**
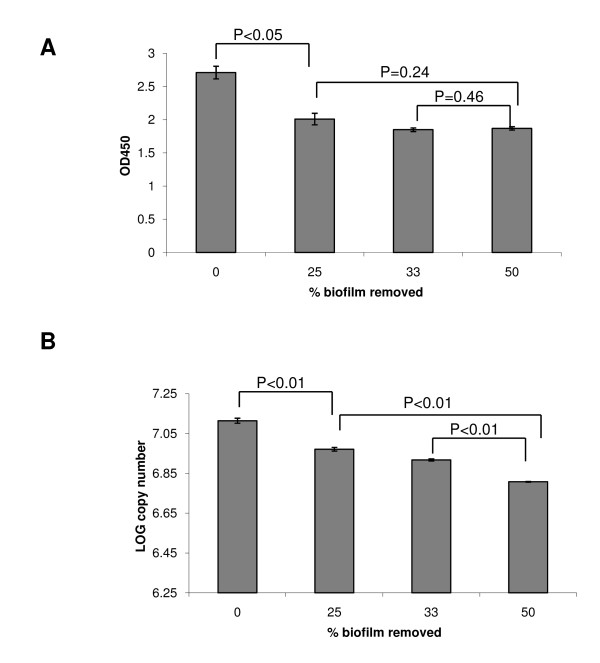
**Comparison of the XTT and qRT-PCR assays in assessing biomass reduction in mature biofilms**. Biofilms were seeded at 10^5 ^cells per 30 mm^2 ^of well surface area and were incubated for 48 h. Prior to assessment, biofilms were either left intact (0), or were mechanically reduced by 25%, 33% or 50%, followed by the XTT assay (A) or qRT-PCR assay (B). Error bars represent SD of triplicate experiments. Student t-test p values are shown on the graph for each set of comparisons.

In order to compare the ability of the XTT and qRT-PCR assays to accurately quantify changes in viable mature biofilms, the biomass of biofilms grown for 48 hours was mechanically reduced and remaining biofilm cells were assessed with the two assays. The XTT assay showed that removal of 25-50% of the biofilm mass resulted in a detectable decrease in OD450 values, compared to intact biofilm. However, there were no significant differences in the XTT signals resulting from removal of different biofilm amounts, thus the XTT signal reduction was not commensurate to the reductions in biomass. This shows that the XTT assay cannot accurately quantify changes in mature biofilms (Figure 5A). In contrast, the qRT-PCR assay showed excellent agreement with reduction in the biofilm mass since 25%, 33% and 50% biofilm removal resulted in an average of 25.8%, 35.4% and 49.8% reduction in the logarithmic *EFB1 *transcript copy numbers, respectively (Figure 5B). This confirms the ability of the real-time RT-PCR assay to accurately measure reduction in biofilm metabolic activity in mature biofilms.

Neutrophils exhibit potent candidacidal activities in vitro [26,27] and interact with *Candida *biofilms forming on mucosal tissues in vivo [4]. However there is a paucity of information regarding the outcome of the interactions of neutrophils with biofilm organisms [28]. One of the difficulties in studying these interactions in vitro is the shortage of quantitative assays to accurately assess neutrophil-inflicted damage in mature biofilms. Therefore, we compared the ability of the two assays to detect and quantify damage inflicted to early and mature biofilms by HL-60 cells, a human neutrophil-like cell line.

**Figure 6:**
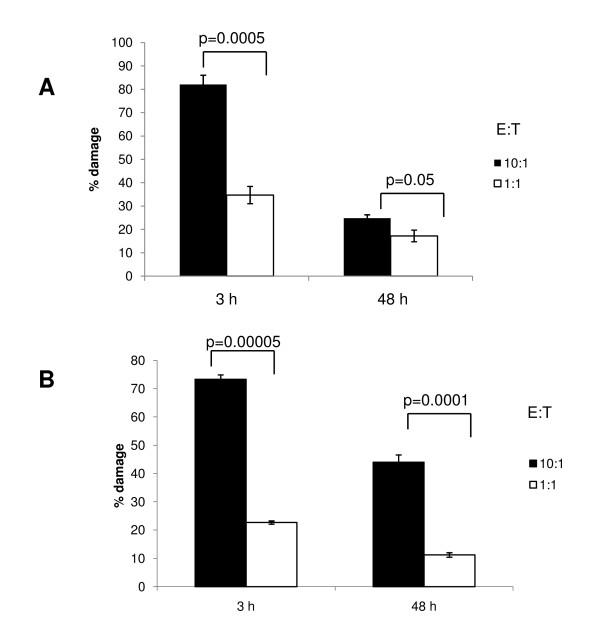
**Comparison of the two assays in quantifying immune effector cell-mediated damage**. Biofilms were seeded at 10^5 ^cells per 30 mm^2 ^of well surface area and were incubated for 3 h or 48 h. HL-60 cells were subsequently added at two E:T ratios (10:1, dark bars; 1:1, light bars). Early or mature biofilm changes were quantified with the XTT (A) or qRT-PCR assays (B). % biofilm damage was calculated using changes in mean OD450 signals or mean *EFB1 *transcript copy numbers, in the presence or absence of effectors, as described in the text. Bars represent SD of triplicate HL-60 experiments. Student-t test p values are shown on the graph for each set of comparisons.

When HL-60 cells interacted with early (3 h) biofilms, significant biofilm damage (up to 80%) could be detected at 10:1 effector to target ratio, regardless of the assay used to measure viable biofilm changes (Figure 6A,B). Significant dose response differences to the number of effectors could also be detected with both assays in early biofilms. Thus there was close agreement between the two assays when early biofilms were tested. However, in mature biofilms the XTT assay revealed overall less than 25% fungal damage by immune effectors and produced similar signals regardless of the number of effectors present, although there was a slight indication of a dose response (Figure 6A). In contrast, the real-time RT-PCR assay revealed a more robust dose response of mature biofilms to immune effectors, with damage to mature biofilms ranging approximately between 10-45%, depending on the effector to target ratio (Figure 6B). Nevertheless, regardless of the assay, early biofilms exhibited significantly higher susceptibility to neutrophil-like cells than mature biofilms, consistent with a recent report [28].

**Figure 7:**
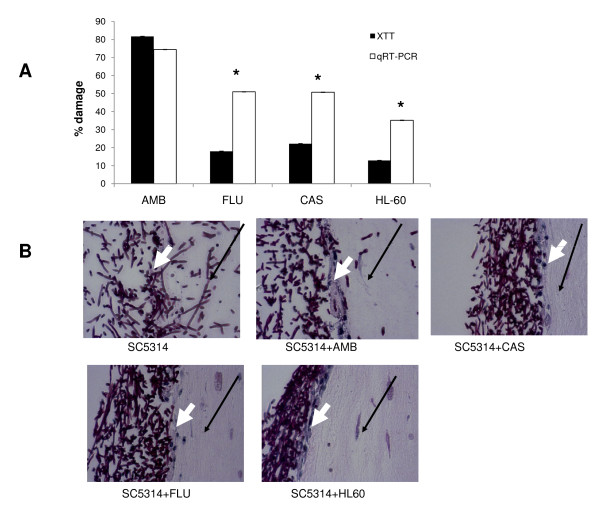
**Biofilm susceptibility testing on a three dimensional oral mucosal culture**. Candida biofilms were grown for 24 h and subsequently exposed to antifungal drugs (4 μg/ml amphotericin B, 70 μg/ml fluconazole or 8 μg/ml caspofungin) or neutrophil-like HL-60 cells at an effector to target cell ratio of 10:1, for 24 additional hours. (A) The effects of antifungal agents on biofilms were quantitatively assessed by the XTT and qRT-PCR assays. Results represent the mean ± SD of one representative experiment where each condition was set up in triplicate. *p < 0.01 for comparison between XTT and qRT-PCR in each condition. (B) PAS stain of histologic sections showing the ability of the biofilm organisms to invade into the submucosal compartment after exposure to antifungal drugs or leukocytes. Black arrows: submucosal compartment. White arrows: epithelial layer. Microorganisms appear in dark blue.

We next compared the performance of the XTT and qRT-PCR assays in quantifying viability changes in mature biofilms grown on a three dimensional model of the human oral mucosa. In order to do this we measured the effects of three antifungal drugs with different mechanisms of action, as well as damage inflicted by human leukocytes to mucosal biofilms. As expected, the data showed that the XTT assay underestimates damage to mature biofilms in this system, when smaller levels of biofilm toxicity are measured, such as the ones obtained with fluconazole, caspofungin or leukocytes (Figure 7A). In contrast, the qRT-PCR assay revealed significant *Candida *toxicity by all antifungal agents tested, which was consistent with the limited levels of *Candida *tissue invasion into the submucosal compartment in the presence of these agents (Figure 7B).

## Discussion

Real-Time PCR technologies combine the sensitivity of conventional PCR with the generation of a quantifiable fluorescent signal and have been increasingly used to assess viability of microorganisms [11,29-31]. Quantitative real-time PCR allows for the detection of PCR products produced at each step of the reaction, since an increase in reporter fluorescent signal is directly proportional to the number of amplicons generated. As we have done in this work, PCR products can be quantitated by generating a standard curve, in which the absolute concentration of the plasmid standard is known.

In this study we measured the effect of anti-fungal agents against mature biofilms with a real-time RT-PCR assay based on the quantification of *EFB1 *transcript copy numbers in biofilm cells. The *EFB1 *gene is constitutively expressed under most growth conditions and is frequently used as a normalization gene in real-time RT-PCR quantification of other *Candida *genes [32-37]. By designing sense primers that span an intron splice site in the *EFB1 *sequence, we expected that only intact mRNA molecules would serve as a template in the RT-PCR assay and that these molecules would be degraded following the death of the organisms in the biofilm. Our results with this molecular assay are consistent with our expectations and show that it is highly quantitative in a wider range of seeding fungal cell densities and that it more accurately measures small-moderate mature biofilm changes in response to stressors, compared to the traditional XTT assay. We have also shown that this assay is particularly well suited for fungal biofilm viability estimates in complex biological systems containing immune effectors or mucosal cell cultures. This may be partly due to the fact that mammalian cells also metabolize XTT, which further limits substrate availability [19].

Compared to the XTT assay, the real-time assay is more technically demanding, more prone to experimental errors due to the multiple additional steps required in sample preparation, more costly, and significantly more time consuming. Thus it should be reserved for susceptibility testing of mature biofilms growing in complex biological model systems containing immune effectors or mucosal cells or used as a confirmatory assay when small changes in mature biofilms are detected with the XTT assay.

## Conclusions

In conclusion, our results indicate that the XTT assay has to be applied with caution to biological systems containing large numbers of organisms alone or in combination with mammalian cells. We also conclude that molecular assessment of biofilms based on quantitation of *EFB1 *transcripts is a sensitive, reproducible and quantitative method to measure the damaging effect of anti-fungal agents against mature biofilms. The new quantitative assay will aid in further investigations of the mechanisms of *Candida *biofilm resistance to immune effector cells, which are presently unknown. In addition the new assay can be applied in quantitative assessment of mature biofilm susceptibility to novel anti-biofilm therapeutic agents that are applied topically on mucosal surfaces.

## Methods

### Biofilm Growth

Strain C. *albicans *SC5314 was used in this study [38]. Yeast from frozen stocks were maintained on YPD agar plates. For experimentation, yeast were inoculated into YPD broth supplemented with 2% dextrose and grown overnight at 24°C with shaking. Biofilms were grown by seeding *C. albicans *blastoconidia in flat bottom well plates (Becton Dickinson, Franklin Lakes, N.J.) and incubating at 37°C from 3 h to 48 h. In preliminary work, five different seeding media (YNB-0.5% glucose, DMEM, DMEM-5%FBS, DMEM-10%FBS and RPMI-10%FBS) were tested. Microscopic observations showed that the best attachment of biofilms to plastic was achieved in DMEM-10%FBS. Thus we used DMEM-10%FBS (Biowest/USA Scientific) in all experiments that followed.

To grow biofilms under conditions resembling in vivo mucosal biofilm development a three dimensional model of the human oral mucosa, developed in our laboratory, was used which faithfully mimics the oral mucosal tissue architecture in vivo [39]. Briefly, this model system is composed of 3T3 fibroblasts embedded in a biomatrix of collagen type I, overlaid by a multilayer of well-differentiated oral epithelial cells (OKF6/TERT-1). *C. albicans *cells (1 × 10^6 ^yeast cells) were added to the cultures apically in 100 μl of airlift medium without FBS and antibiotics and incubated for 24 h. To assess mucosal tissue damage and invasion tissues were formalin-fixed, embedded in paraffin and 5 μm sections were stained with the Periodic Acid Schiff (PAS) stain.

### XTT Assay

The XTT assay was performed as we described earlier [7]. Briefly, media were aspirated from biofilms and were replaced with 100 μl/well of XTT solution (Sigma Chemical Co., St. Louis, MO) containing Coenzyme Q0 (CoQ, Sigma Chemical Co., St. Louis, MO). Fresh mixtures of XTT and CoQ [1 mg/ml and 40 μg/ml (or 220 μM), respectively, unless otherwise indicated] were prepared for each experiment. Plates were incubated at 37°C for up to 3 hours, after which supernatants were transferred into new plates, and optical densities (OD) were measured by an Opsys Microplate Reader (Thermo Labsystems, Franklin, MA) at 450-490 nm, with a 630 nm reference filter. When optical densities were higher than the limits of the microplate reader, dilutions of the supernatants in water were made.

### Quantitative Real-Time RT-PCR Assay

To quantify changes in viable biofilms using an alternative approach, we measured mRNA expression of the translation elongation factor-1β (EF-1β), encoded by the *EFB1 *gene in *C. albicans*, by real-time quantitative RT-PCR. The choice of this gene was based on these facts: a) fungal cells transcriptionally regulate components of their translational machinery according to external stresses that affect protein synthesis [25,36]; b) the number of *EFB1 *gene transcripts did not change significantly during biofilm or planktonic growth between 3 h and 48 h (not shown); and c) *EFB1 *is frequently used as a housekeeping gene in real-time RT-PCR quantification of other *Candida *genes [29,32,33,36]. Total RNA from biofilms was isolated using the RiboPure yeast kit (Ambion, Inc.), according to the manufacturer's instructions. RNA concentrations and purity were determined by measuring the absorbance at 260 nm and 280 nm (ND-1000 spectrophotometer, NanoDrop Technologies). Equal amounts of RNA (3 μg in 20 μl reactions) were reverse transcribed with oligo(dT) primers using Superscript reverse transcriptase II (Invitrogen).

Primers were based on the published sequence of the *EFB1 *gene of *C. albicans*. Primer sequences used were as follows: Forward: 5'- CAT TGA TGG TAC TAC TGC CAC -3'; Reverse: 5'- TTT ACC GGC TGG CAA GTC TT -3'. The forward primer spanned the sole exon-exon boundary of *EFB1*, thus excluding amplification of genomic *C. albicans *DNA. The uniqueness of the primers for *C. albicans EFB1 *was determined using the BLAST database http://www.tigr.org.

To generate standard curves for quantitative analyses a pEFB plasmid was prepared as follows. A 136-bp *C. albicans EFB *exon fragment, containing the target sequence, was amplified with the above mentioned primers. PCR was performed in a DNA thermal cycler with 1 cycle of 5 min at 95°C; 40 cycles of 1 min at 95°C, 30 s at 62°C, 30 s at 72°C; and a final extension at 72°C for 5 min. This fragment was ligated into the pCR 2.1 plasmid vector (3.931 kb) and transformed into One Shot cells (Top10F') using a TA cloning kit (Invitrogen). Plasmids were digested with *xho*I to generate a linear template and purified with the PureYield™ Plasmid Miniprep System (Promega). Plasmid concentrations were determined spectrophotometrically and copy numbers calculated based on linear plasmid mass. Serial plasmid dilutions (500 pg, 50 pg, 5 pg, 500 fg, 50 fg, 5 fg, 1 fg of DNA/μl) were then used to generate standard curves for detection and quantification of EFB1 mRNA by the iCycler iQ RT-PCR assay.

Real-time PCR was performed with an iCycler iQ real-time PCR detection system (Bio-Rad). All PCR reaction mixtures contained the following: 10 μl 2 × iQ™ SYBR^® ^Green Supermix (BioRad, Hercules, CA), 1 μl of first-strand cDNA reaction mixture or linear plasmid DNA, 0.1 μM of primers and H_2_O to bring the final volume to 20 μl. The program for amplification had an incubation step at 50°C for 2 min, and 95°C incubation for 5 min, followed by 40 cycles of 95°C for 10 s and 62°C for 30 s. Reactions to estimate transcript copy number were run in duplicate from two biologic RNA replicates. Data were analyzed using the iCycle iQ system software (BioRad).

### Testing of planktonic cells

Candida cells were grown overnight in YPD broth as described above. Cultures were adjusted to a cellular density equivalent to 1.0 × 10^6 ^cells/ml and subjected to caspofungin (CAS, Merck Research Laboratories, Rahway, N.J.) or fluconazole (FLU, Pfizer Inc., Sandwich, United Kingdom) treatment for 24 h, at doses ranging from 0.0625-1024 μg/ml [40,41]. Untreated cells served as negative controls. Four replicates were included in each experiment. The effects of the anti-fungals on planktonic cells were measured by colony counts on Sabouraud agar plates (CFU), or by the XTT and qRT-PCR assays as described above.

### Biofilm testing

To compare the ability of the two assays to quantify changes in mature biofilms stemming from biomass reduction, organisms were grown in 12 well plates for 48 h and their biomass was physically reduced by removing 50%, 33% or 25% of the biofilm from the well surface. To perform this, the round surface area of each well was divided into two, three or four equal parts, and removal of the biofilm from 1/2, 1/3 or 1/4 of the surface area was accomplished with the help of a modified rubber policeman, with a sweeping edge cut to the size of the well radius. Remaining biofilm cells observed microscopically were removed using a sterile glass suction tip. XTT and real-time RT-PCR measurements in residual biofilms in these wells were subsequently compared to intact biofilms.

To compare the ability of the two assays to quantify changes in viable biofilms in response to different stressors, biofilms grown on plastic were exposed to pharmacologic [amphotericin B (AMB), 4 μg/ml, 4 h], environmental (100°C, 1 h) or immune cell stressors and viability was measured by the XTT or qRT-PCR assays. To quantify susceptibility to immune cell-inflicted damage we used a neutrophil-like cell line (HL-60, ATCC), as previously described [7]. Briefly, pre-activated HL-60 cells (1.25% DMSO for 7-9 days) were added to biofilms at varying effector to target cell ratios, based on seeding cell densities. After incubation at 37°C, 5% CO_2 _for 2 hours, media were aspirated, HL-60 cells were lysed with sterile H_2_O, and fungal viability was assessed with the XTT or qRT-PCR assays. Biofilms grown on mucosal tissues were exposed to anti-fungal drugs (4 μg/ml amphotericin B, 70 μg/ml fluconazole or 8 μg/ml caspofungin [40,41]) or HL-60 cells for 24 hours, followed by mammalian cell lysis with sterile water. This was followed by the XTT or qRT-PCR assays. Anti-biofilm activity was calculated according to the following formula: % fungal damage = (1-x/n)*100, where × is the OD450 or *EFB1 *transcript copy number of experimental wells (*C. albicans *with stressors/effectors) and n is the OD450 or *EFB1 *transcript copy number of control wells (*C. albicans *only). All experiments were performed in triplicate.

## Competing interests

The authors declare that they have no competing interests.

## Authors' contributions

ZX participated in the design of the study, performed the experimental procedures, carried out the data analysis, and drafted the manuscript. AT and HK helped in certain experimental procedures. ADB conceived the study and helped to draft the manuscript. All authors read and approved the final manuscript.
